# Genome-wide identification of genes encoding SWI/SNF components in soybean and the functional characterization of *GmLFR1* in drought-stressed plants

**DOI:** 10.3389/fpls.2023.1176376

**Published:** 2023-05-15

**Authors:** Qiang Chen, Xiaolei Shi, Lijuan Ai, Xuan Tian, Hongwei Zhang, Jiawang Tian, Qianying Wang, Mengchen Zhang, Sujuan Cui, Chunyan Yang, Hongtao Zhao

**Affiliations:** ^1^ Hebei Key Laboratory of Molecular and Cellular Biology, Key Laboratory of Molecular and Cellular Biology of Ministry of Education, Hebei Research Center of the Basic Discipline of Cell Biology, Hebei Collaboration Innovation Center for Cell Signaling, College of Life Science, Hebei Normal University, Shijiazhuang, Shijiazhuang, Hebei, China; ^2^ Hebei Laboratory of Crop Genetics and Breeding, National Soybean Improvement Center Shijiazhuang Sub-Center, Ministry of Agriculture and Rural Affairs, Huang-Huai-Hai Key Laboratory of Biology and Genetic Improvement of Soybean, Institute of Cereal and Oil Crops, Hebei Academy of Agricultural and Forestry Sciences, Shijiazhuang, Hebei, China

**Keywords:** soybean, SWI/SNF components, GmLFR1, transgenic hairy root, drought stress

## Abstract

ATP-dependent SWI/SNF chromatin remodeling complexes (CRCs) are evolutionarily conserved multi-component machines that regulate transcription, replication, and genome stability in eukaryotes. SWI/SNF components play pivotal roles in development and various stress responses in plants. However, the compositions and biological functions of SWI/SNF complex subunits remain poorly understood in soybean. In this study, we used bioinformatics to identify 39 genes encoding SWI/SNF subunit distributed on the 19 chromosomes of soybean. The promoter regions of the genes were enriched with several *cis*-regulatory elements that are responsive to various hormones and stresses. Digital expression profiling and qRT-PCR revealed that most of the SWI/SNF subunit genes were expressed in multiple tissues of soybean and were sensitive to drought stress. Phenotypical, physiological, and molecular genetic analyses revealed that *GmLFR1* (*Leaf and Flower-Related1*) plays a negative role in drought tolerance in soybean and *Arabidopsis thaliana*. Together, our findings characterize putative components of soybean SWI/SNF complex and indicate possible roles for GmLFR1 in plants under drought stress. This study offers a foundation for comprehensive analyses of soybean SWI/SNF subunit and provides mechanistic insight into the epigenetic regulation of drought tolerance in soybean.

## Introduction

Soybean (*Glycine max*) is an economically and nutritionally essential leguminous crop, which is widely used for vegetable protein and edible oil. Legumes can grow in a broad range of climates, but they are sensitive to abiotic environmental stresses (Hossain et al., 2013). For example, drought limits the growth, development, and yield of soybean, causing adverse agricultural and economic losses worldwide (Anderson et al., 2019; Zhang et al., 2021). The appropriate regulation of gene expression in the face of environmental stresses is critical for crop survival and yields (Song et al., 2021). The characterization of functional genes that are involved in stress tolerance is thus of pivotal significance to ensure sustainable soybean production (Leng et al., 2021). Several studies have yielded important insight into the functions of various soybean transcription factors, including soybean Nuclear Factor-Y (GmNF-Ys), WRKY-type transcription factor (GmWRKYs), GmMADSs, Drought response element binding (GmDREBs), GmMYBs, Basic leucine zipper (GmbZIPs), GmNACs (NAM, ATAF and CUC) and Late elongated hypocotyl (GmLHYs) transcription factors, in drought tolerance, and several drought-related quantitative trait loci and candidate genes have been identified (Hussain et al., 2017; Wei et al., 2019; Zhang et al., 2019; Ma et al., 2020; Wang et al., 2020; Yang et al., 2020; Chen et al., 2021; Yu et al., 2021; Zhang et al., 2022). Epigenetic factors, including chromatin remodeling, DNA methylation, and histone modification, play pivotal roles in the regulation of gene expression in plants under conditions of drought stress (Song et al., 2021). However, the epigenetic regulators that function in drought-stressed soybean plants, especially chromatin remodeling complexes (CRCs), are largely unknown.

ATP-dependent CRCs are crucial epigenetic factors that regulate gene transcription by altering chromatin or nucleosome conformation using the energy generated by ATP hydrolysis (Holde and Yager, 2003; Clapier and Cairns, 2009). Switch defective/sucrose non-fermentable (SWI/SNF) complexes are well-known CRCs that are conserved throughout eukaryotes (Clapier et al., 2017). In the model plant Arabidopsis, more than 19 SWI/SNF subunits have been identified that have diverse roles in plant growth and development, including four ATPase catalytic subunits [BRAHMA (BRM), SPLAYED (SYD), chromatin remodeling factor (CHR12), and CHR23], four SWI3 subunits (SWI3A-SWI3D), two SWP73 subunits (SWP73A and SWP73B), two actin-related proteins (ARP4 and ARP7), one SNF5 subunit, two BRM-interacting proteins (BRIP1 and BRIP2), three bromodomain-containing proteins (BRD1, BRD2, and BRD13), and one Leaf and Flower-Related (LFR) subunit (Wagner and Meyerowitz, 2002; Farrona et al., 2004; Mlynárová et al., 2007; Brzeski J et al., 1999; Sarnowski et al., 2005; Han et al., 2015; Lin et al., 2018; Lin et al., 2021; Shang and He, 2022; Yu et al., 2021). SWI/SNF subunits are also involved in a variety of abiotic stress responses in plants. For example, in Arabidopsis, BRM binds directly to the promoter of ABA INSENSITIVE 5 and represses its transcription in response to abscisic acid (ABA) and drought stress (Han et al., 2012), and the loss-of-function mutant of *CHR12* shows less growth arrest under stress conditions (Mlynárová et al., 2007; Leeggangers et al., 2015). Moreover, we found that *lfr* mutants are hypersensitive to salt stress (Yang et al., 2016), whereas the *brm-5* mutant is able to tolerate high-boron stress because 26S proteasome subunits promote the degradation of BRM and thus limit chromatin opening by BRM to maintain chromatin stability and avoid DNA double-strand break formation after boron exposure (Sakamoto et al., 2018). Meanwhile, numerous studies have revealed the complex relationship between SWI/SNF subunits and multiple hormones, including ABA, ethylene (ET), and jasmonic acid (JA), suggesting the involvement of SWI/SNF complex in multiple abiotic and biotic stress responses in Arabidopsis (Sarnowska et al., 2016). In addition, the functions of SWI/SNF components are gradually being uncovered in other crops. For instance, OsSWI3C plays a negative role in drought resistance in rice by suppressing the expression of drought resistance- or root growth-related genes (Yang et al., 2020), whereas the chromatin remodeler ZmCHB101 (an ortholog of AtSWI3D) interacts with RNA polymerase II to directly affect the expression of stress-responsive genes and regulate osmotic and dehydration stress responses in maize (Yu et al., 2016; Yu et al., 2018).

Here, we report the genome-wide identification of SWI/SNF subunits by *in silico* analyses of gene structures and protein properties, evolutionary relationships, and putative promoter elements. The spatiotemporal and drought-induced expression patterns of several SWI/SNF subunit genes were also studied. We found that the SWI/SNF subunit GmLFR1 is involved in drought stress responses using transgenic soybean and Arabidopsis plants. Our results provide bioinformatic and experimental bases for identifying candidate SWI/SNF subunits with important roles in the drought response of soybean.

## Materials and methods

### Bioinformatic analysis of SWI/SNF components in the soybean genome

The genome and protein sequences of the soybean cultivar “Williams 82” (Glycine max Wm82.a4.v1) were downloaded from Phytozome 13 (https://phytozome.jgi.doe.gov/pz/). SWI/SNF subunit protein sequences identified in Arabidopsis and *Oryza sativa* were downloaded from TAIR (http://www.arabidopsis.org) and the rice data center of China (http://www.ricedata.cn/gene/index.htm), respectively. For the identification of SWI/SNF subunits in soybean, we searched the soybean genome database using the HMM profiles in TBtools-II v1.108 to carry out a BLAST-P with 1e-5 set as the *E*-value (Chen et al., 2020). The physicochemical parameters of the SWI/SNF subunits of soybean were determined using ExPASy-ProtParam (http://web.expasy.org).

The chromosomal locations, intron numbers, and sizes (bp) of soybean SWI/SNF complex subunit genes were obtained using Phytozome 13. The exon/intron structures of the SWI/SNF subunit genes in soybean were analyzed using TBtools software (Chen et al., 2020). Conserved domains of the soybean and Arabidopsis SWI/SNF component genes were predicted using SMART (http://smart.embl.de/).

The promoter sequence (about 2,000 bp upstream of the start codon, ATG) of each SWI/SNF component gene predicted in this study was extracted from the *Glycine max* Wm82.a4.v1 genome sequence using TBtools software. *Cis*-regulatory elements in the sequences were analyzed using the PlantCARE database (Lescot et al., 2002).

The amino acid sequences of the SWI/SNF subunits from Arabidopsis, rice, and soybean were aligned using ClustalW. A phylogenetic tree was constructed using MEGA version 7.0 with the neighbor-joining method and a bootstrap of 1,000 replicates (Kumar et al., 2016).

To dissect the expression patterns of the SWI/SNF subunit genes in soybean, we obtained transcriptome data from 1,248 libraries available in the Soybean Expression Atlas (http://venanciogroup.uenf.br/resources/). We processed the transcripts per million values and generated a heatmap. Gene-wise normalization of the expression data and creation of the heatmap were done using TBtools (Chen et al., 2020).

### Plant materials and growth conditions

Soybean cultivar *Williams* 82 was used for gene expression pattern analysis and *Agrobacterium rhizogenes*-mediated hairy root transformation. Soybean seedlings were cultured in a temperature-controlled chamber (26°C, 16 h of light/8 h of dark, and 60% relative humidity). The Arabidopsis *lfr-2* mutant [ecotype Columbia (Col-0)] is described elsewhere (Wang et al., 2009). Arabidopsis plants were cultured at 22°C in a greenhouse under long-day conditions (16 h of light/8 h of dark).

### Transgenic plant production and drought stress assays in Arabidopsis

The full-length coding sequence (CDS) of *GmLFR1* without the stop codon was amplified by PCR with the primers listed in **Supplementary Table S1** from *Williams* 82 cDNA, and then cloned into pCAMBIA1300 under the control of the cauliflower mosaic virus (CaMV) 35S promoter and fused with the green fluorescence protein gene (*GFP*) (creating *35S*:*GmLFR1-GFP*) using *Xba*I and *Bam*HI. After verification by DNA sequencing, the binary vector was transformed into *Agrobacterium tumefaciens* GV3101 and transformed in *lfr-2*/+ using the floral dip method. Transformants were selected on 1/2 strength Murashige and Skoog medium containing 50 mg/L of hygromycin; T3 plants were used for further analysis. To assay for drought stress in Arabidopsis, the Col-0, *lfr-2*, and *35S*:*GmLFR1-GFP/lfr-2* seedlings planted into the soil were watered regularly to allow them to grow to three weeks old, and they were watered for the last time then. In the following days, the plants were gradually subjected to drought stress by withholding water for three weeks and photographed. Survival rate (%) was measured 5 days after re-watering. The malonaldehyde (MDA) content of leaves from Col-0, *lfr-2*, and *35S*:*GmLFR1-GFP/lfr-2* plants with or without drought treatment were determined using the corresponding kits according to the manufacturer’s protocols (Cominbio, Suzhou, China). For relative water loss, rosette leaves of 3-week-old Col-0, *lfr-2*, and *35S*:*GmLFR1-GFP/lfr-2* plants were detached and placed on a bench at room temperature for 5.5 h, and the fresh weights of the leaves were measured every 0.5 h.

### *Agrobacterium rhizogenes*-mediated hairy root transformation and drought assays in soybean

The CDS of *GmLFR1* without the stop codon was amplified using the primers listed in **Supplementary Table S1**, and then cloned into the binary vector pUB-GFP with the addition of p*Ubi*:3×*Flag* (empty vector, EV) for gene overexpression (Zhang et al., 2021) to obtain p*Ubi : GmLFR1-*3×*Flag* (*GmLFR1*-OE) using *Kpn*I. Following confirmation by DNA sequencing, the *GmLFR1*-OE and EV vectors were transferred into *A. rhizogenes* strain K599 and then injected into hypocotyls as described previously (Kereszt et al., 2007; Du et al., 2018). The injected plants were placed in a greenhouse and kept at high humidity until hairy roots were generated at the infection site and had grown to about 5 cm in length. The hypocotyl was then removed at about 1 cm below the infected site. The seedlings were then transplanted into water in a greenhouse for 3 days and then transplanted into soil for 1 week. Next, the seedlings were transferred to 10% (w/v) polyethylene glycol (PEG)-6000 for stress treatment. The leaves of *GmLFR1*-OE and EV-control seedlings with or without PEG-6000 treatment were obtained to measure physiological indicators. The catalase (CAT), peroxidase (POD), and superoxide dismutase (SOD) activity levels and the MDA contents of the leaves were determined using the corresponding kits according to the manufacturer’s protocols (Cominbio).

### Expression analysis of SWI/SNF component genes and drought-responsive genes in soybean

To determine the tissue-specific expression patterns of SWI/SNF components in soybean, the terminal buds, leaves, roots, and flowers of *Williams* 82 plants grown under normal conditions were sampled and frozen in liquid nitrogen for RNA extraction. To determine the expression patterns in drought-treated plants, 3-week-old seedlings were treated with 8% PEG-6000 for 6 h, 12 h, and 24 h, respectively. Mock-treated plants were used as a control. Then, the leaves of the control and treated seedlings were sampled and frozen in liquid nitrogen for RNA extraction. To detect the expression of drought-responsive genes, the roots of GmLFR1-OE and EV-control seedlings with or without drought treatment for 24 h were isolated and frozen in liquid nitrogen for RNA extraction. Total RNA isolation was done using a FastPure^®^ Plant Total RNA Isolation Kit (Vazyme, Nanjing, China). Then, 1,000 ng of RNA were used for cDNA synthesis with HiScript^®^ IIQRT SuperMix (Vazyme). Specific pairs of primers for the soybean SWI/SNF subunit genes were designed using Primer Premier 5.0 (**Supplementary Table S1**). Quantitative real-time RT-PCR (qRT-PCR) was conducted using an Applied Biosystems StepOnePlus™ Real-Time System (Waltham, MA, USA). *ACTIN2* was used as the internal reference gene (Le et al., 2011). Each experiment was performed with three biological replicates. The 2^−ΔΔCт^ method was used to evaluate the relative expression levels of different genes (Livak and Schmittgen, 2001).

### Subcellular localization assays

The roots of 6-day-old *35S*:*GmLFR1-GFP/lfr-2* transgenic Arabidopsis seedlings were stained with propidium iodide (PI) or 4’,6-diamidino-2-phenylindole (DAPI) and then imaged using an Olympus FV3000 laser-scanning confocal microscope (Olympus Corp., Tokyo, Japan). The excitation and emission wavelengths of the DAPI/GFP/PI signals were 405/488/543 nm and 430–480/500–550/550–630 nm, respectively.

## Results

### Identification of SWI/SNF components in soybean

To identify SWI/SNF complex members in soybean, 19 protein sequences of Arabidopsis SWI/SNF components, including 4 core enzyme and 15 other subunits, were used as queries and a genome-wide search was carried out using BLAST. A total of 39 SWI/SNF subunit genes were identified in the soybean genome (**Table 1**). Most Arabidopsis SWI/SNF subunit genes had two to four orthologs, except for *AtSNF5*, which had only one ortholog, in the soybean genome. The gene information and protein properties of the soybean SWI/SNF components are shown in **Table 1**. GmSYD2 was the largest protein, with 3,789 amino acids and a molecular weight (MW) of 410.71 kDa, while GmSNF5 was the smallest subunit (27.39 kDa) with 240 amino acids. The MWs of GmLFR1 and GmLFR2 were 50.23 and 53.65 kDa with 460 and 491 amino acids, respectively. The theoretical isoelectric point (pI) of these SWI/SNF subunits ranged from 4.68 (GmARP7B) to 9.56 (GmSWP73B).

**Table 1:** Characterization of the SWI/SNF components in soybean.

Gene	Gene ID	Chr	Gene location (bp)	Exons/Introns	Amino acids (aa)	MW (kDa)	pI
*GmSYD1*	Glyma.07G252100	7	42958598.42982576	36/35	3477	378.26	5.45
*GmSYD2*	Glyma.17G022300	17	1612607.1636461	36/35	3789	410.71	5.16
*GmBRM1*	Glyma.16G035100	16	3320481.3332717	14/13	2203	246.59	9.17
*GmBRM2*	Glyma.07G069400	7	6270341.6283104	14/13	2226	249.56	9.06
*GmBRM3*	Glyma.18G234700	18	52303924.52315500	14/13	2222	247.58	8.81
*GmBRM4*	Glyma.09G257900	9	47674488.47686619	14/13	2222	247.62	8.67
*GmMINU1*	Glyma.10G250500	10	47849565.47859618	12/11	1072	123.74	7.94
*GmMINU2*	Glyma.20G143200	20	38178122.38190120	12/11	1073	123.87	7.02
*GmMINU3*	Glyma.11G004100	11	306360.313607	12/11	1063	122.74	7.14
*GmLFR1*	Glyma.02G001600	2	182962.186963	8/6	460	50.23	6.24
*GmLFR2*	Glyma.10G001900	10	203415.207372	8/6	491	53.65	8.42
*GmARP4A*	Glyma.03G107300	3	30703105.30720374	19/18	445	48.9	5.04
*GmARP4B*	Glyma.07G118800	7	13519273.13538642	19/18	446	49.06	5.14
*GmARP4C*	Glyma.08G040300	8	3171371.3178118	19/18	431	47.63	5.25
*GmARP7A*	Glyma.15G275500	15	51432808.51446774	7/6	361	39.55	4.82
*GmARP7B*	Glyma.12G007600	12	558537.562408	7/6	361	39.4	4.68
*GmARP7C*	Glyma.10G089200	10	11895977.11919750	7/6	361	39.49	4.78
*GmARP7D*	Glyma.09G229000	9	45307748.45311519	7/6	330	36.11	4.73
*GmSNF5*	Glyma.13G117200	13	23041213.23044580	10/9	240	27.39	5.37
*GmSWI3A1*	Glyma.04G243100	4	51117576.51121842	7/6	527	58.93	5.07
*GmSWI3A2*	Glyma.06G120200	6	9773538.9777762	7/6	523	58.43	5.13
*GmSWI3B1*	Glyma.04G014400	4	1109400.1113097	5/4	484	53.79	5.8
*GmSWI3B2*	Glyma.06G014400	6	1088340.1091991	5/4	491	54.91	5.8
*GmSWI3C1*	Glyma.04G247200	4	51433735.51440939	9/8	785	86.44	5.9
*GmSWI3C2*	Glyma.06G115600	6	9407067.9413440	9/8	785	86.76	5.78
*GmSWI3C3*	Glyma.13G031300	13	10193948.10199845	9/8	765	83.87	5.95
*GmSWI3C4*	Glyma.14G153000	14	33274632.33280444	9/8	776	85.35	6.02
*GmSWI3D1*	Glyma.12G044200	12	3205310.321240	7/7	1016	109.93	5.05
*GmSWI3D2*	Glyma.11G118900	11	9028385.9035480	7/7	1047	113.19	5
*GmSWP73A*	Glyma.05G077800	5	10321683.10325195	2/1	543	60.05	9.55
*GmSWP73B*	Glyma.19G071500	19	24514083.24517178	2/1	543	60.09	9.56
*GmBRIP1*	Glyma.12G014000	12	1010585.1015652	2/1	368	42.32	4.97
*GmBRIP2*	Glyma.09G223000	9	44354614.44359768	2/1	378	43.57	5.02
*GmBRD1*	Glyma.04G016900	4	1276681.1281412	9/8	649	71.99	6.63
*GmBRD2*	Glyma.06G017300	6	1277337.1281980	9/8	665	73.57	7.12
*GmBRD13A*	Glyma.08G318100	8	43749007.43755314	9/8	1000	110.64	6.30
*GmBRD13B*	Glyma.08G318200	8	43757207.43763451	9/8	857	95.32	5.99
*GmBRD13C*	Glyma.18G095200	18	9732437.9738098	9/8	867	96.05	5.93
*GmBRD13D*	Glyma.18G095400	18	9748626.9755084	9/8	857	94.50	6.26

Chr, chromosome; MW, molecular weight.

A chromosome-wise map of the soybean SWI/SNF subunit genes was drawn based on the soybean genome database. The 39 soybean SWI/SNF subunit genes were unevenly spaced on the 19 chromosomes. (**Figure S1**; **Table 1**). Specifically, four SWI/SNF subunit genes were located on Chr 04 and Chr 06; only one to three SWI/SNF component genes were located on the other chromosomes except for Chr 01, which had none (**Figure S1**). We found that two pairs of BRD family members, BRD13A/BRD13B and BRD13C/BRD13D, were tandemly arranged in the 14.44-kb region of Chr 08 and the 22.65-kb region of Chr 18, respectively (**Figure S1**). Other paralogous genes were distributed on different chromosomes; for example, *GmLFR1* and *GmLFR2* were located on Chr 02 and Chr 10, respectively (**Figure S1**).

### Gene structure and protein domain analyses of soybean SWI/SNF subunits

**Figure 1:**
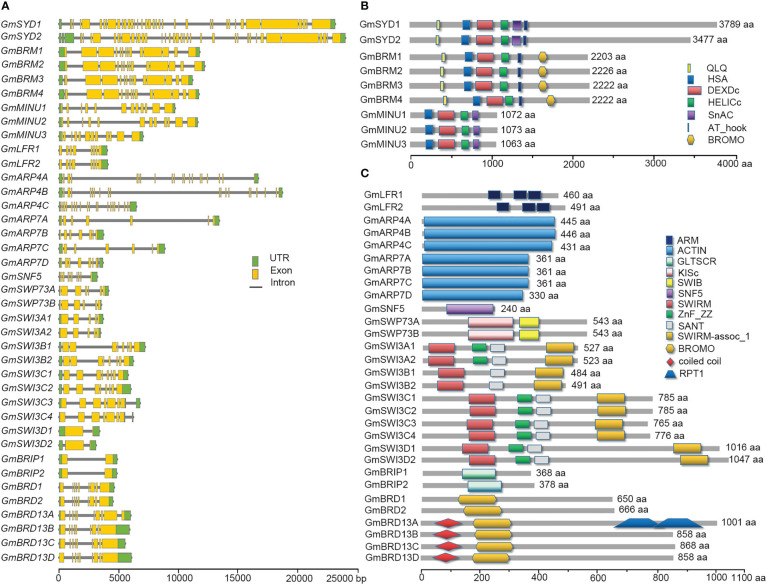
Gene structures and protein domains of the soybean SWI/SNF components. **(A)** Schematic diagrams indicate the structures of genes encoding SWI/SNF components. Introns, exons, and untranslated regions (UTRs) are indicated by gray lines, yellow boxes, and green boxes, respectively. **(B, C)** Schematic diagrams indicate the protein domains of the SWI/SNF complex subunits. Different domains are indicated by different colored shapes. Scale bars for the gene and protein sizes are shown at the bottom.

To further examine the organization of the SWI/SNF components in soybean, gene structures were constructed according to the corresponding genomic sequences and coding sequences (CDSs) using TBtools. The numbers of introns and exons varied greatly among different SWI/SNF subunits (**Figure 1A**). Among the ATPase-encoding genes, each *GmSYDs* contained 36 exons while each *GmBRMs* and *GmMINUs* contained 14 and 12 exons, respectively. The number of exons in the genes encoding the other subunits ranged from 2 (*GmSWP73s* and *GmBRIPs*) to 19 (*GmARP4s*). The protein domains of the SWI/SNF subunits were analyzed using the SMART website. Conserved domains were found between the subunits and their orthologs in Arabidopsis (**Figures 1B, C**). For example, the SWI/SNF ATPase subunits GmSYDs, GmBRMs, and GmMINUs all contained DEAD-like helicases N-terminal domain (DEXDc) and helicase superfamily C-terminal domain (HELICc); GmBRMs also contained a bromodomain; GmLFRs contained Armadillow domains (ARM); GmARPs contained an ACTIN domain; and GmSWI3s contained SWIRM, SANT, and SWIRM-assoc_1 domains.

### Phylogenetic analysis of soybean SWI/SNF components

**Figure 2:**
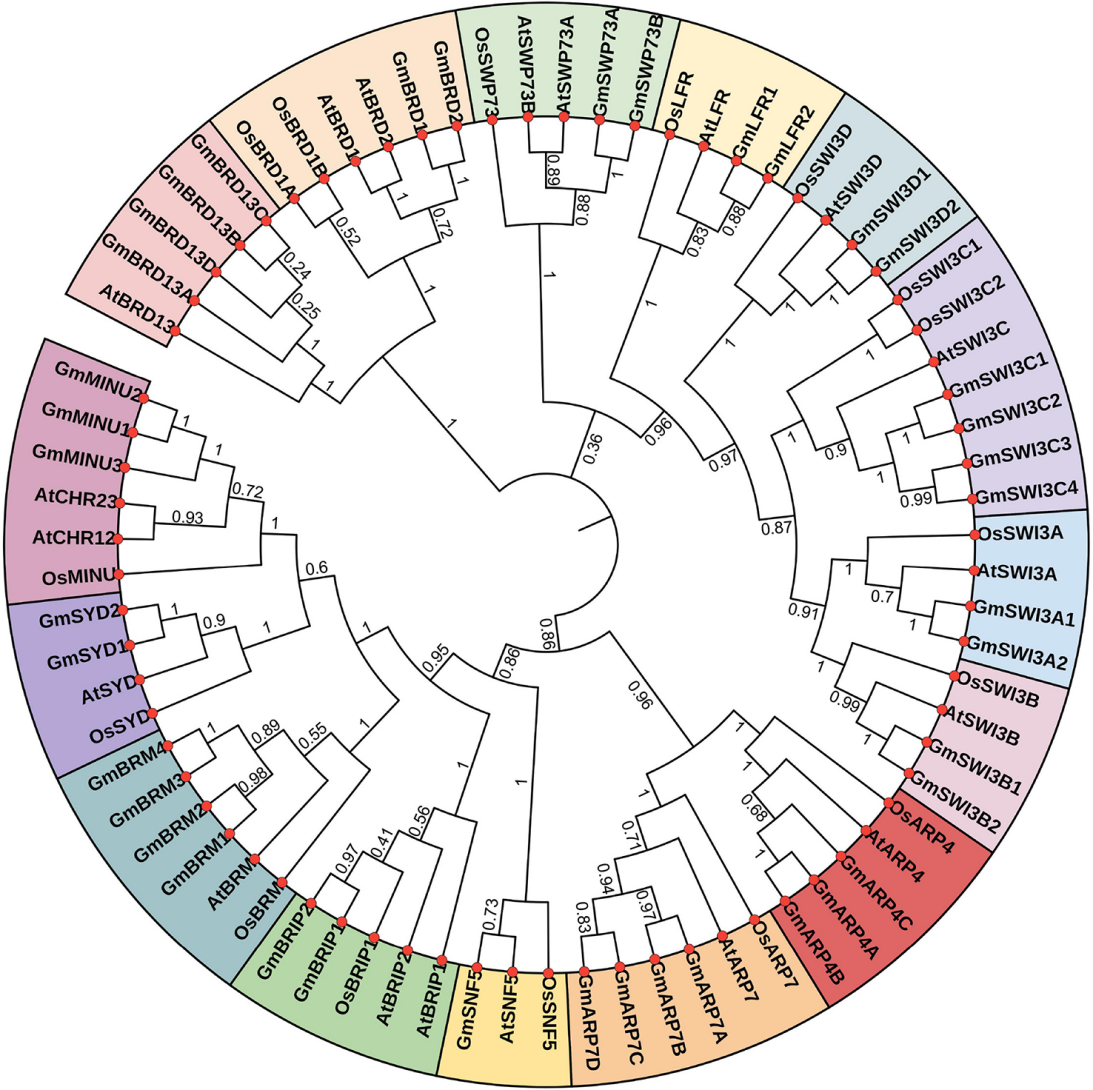
Phylogenetic tree showing the SWI/SNF components from soybean (Gm), rice (Os), and Arabidopsis (At). The tree was constructed using MEGA 7.0. The 15 clusters are represented by different colors.

To study the phylogenetic relationships among SWI/SNF components, a phylogenetic tree was built based on the full-length amino acid sequences of 74 SWI/SNF subunits from soybean (39), rice (16), and Arabidopsis (19) using the Neighbor-Joining method in MEGA7. The 74 SWI/SNF subunit genes were divided into 15 clusters (**Figure 2**). Members of the same subunits in soybean, rice, and Arabidopsis were found to cluster together. Further, most soybean SWI/SNF subunits had a closer evolutionary relationship with their orthologs in Arabidopsis than with those in rice (**Figure 2**).

### *Cis*-regulatory element analysis of the putative promoters of the genes encoding SWI/SNF subunits in soybean

**Figure 3:**
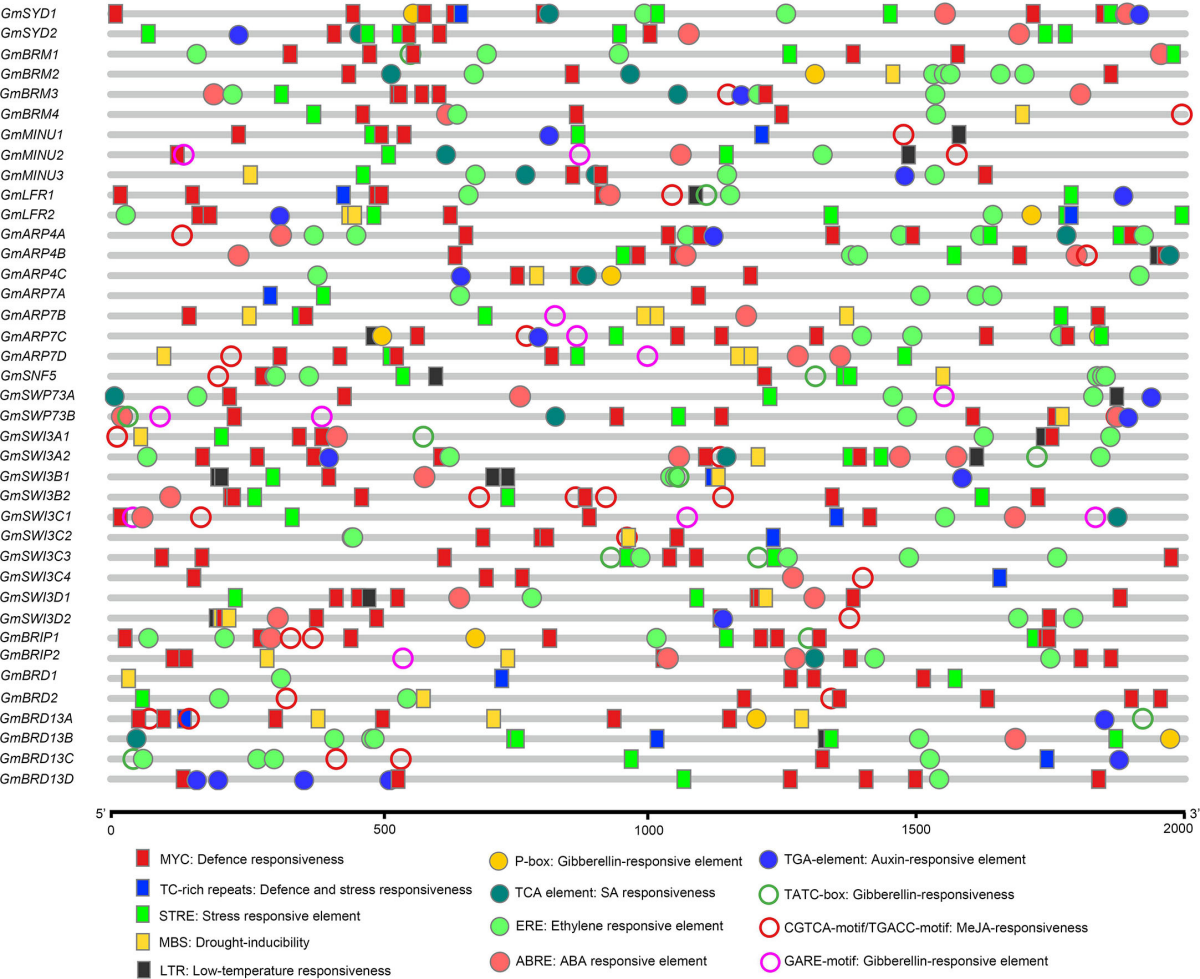
*In silico* analysis of *cis*-regulatory elements in the putative regulatory regions (promoters) of the soybean SWI/SNF component genes. A legend showing the symbols representing the *cis*-regulatory elements along with their corresponding names is given at the bottom.

To determine the potential biological processes and regulatory networks that the soybean SWI/SNF subunits are involved in, we used the PlantCARE database to identify *cis*-regulatory elements in the putative promoters of the identified genes. A series of *cis*-elements were identified in the ~2.0-kb sequences upstream of the ATG start codon in the SWI/SNF component genes (**Figure 3**). The putative promoter regions of the genes included *cis*-elements related to abiotic stress, including MBS (drought-inducible), MYC (drought- and cold-responsive), STRE (stress-responsive element), TC-rich repeats (defense and stress responsiveness), and LTR (low-temperature responsiveness) elements. Furthermore, some phytohormone-responsive elements were identified in the soybean SWI/SNF complex genes, including ABRE (ABA-responsive element), ERE (ET-responsive element), TGA-elements (auxin-responsive element), TCA-elements (salicylic acid-responsive element), and GARE-motifs (gibberellin-responsive element). Thus, the soybean SWI/SNF components we identified may be involved in abiotic stress responses and the regulation of biological processes.

### Expression pattern of soybean SWI/SNF components

**Figure 4:**
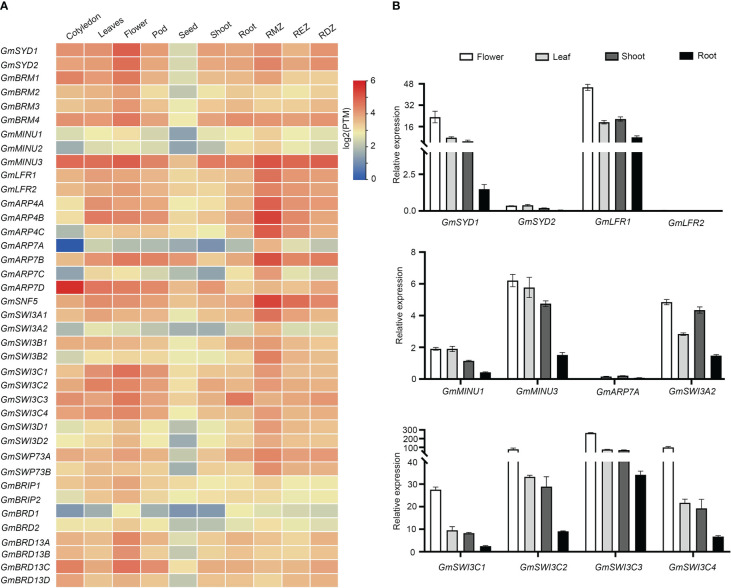
Transcriptional expression levels of soybean SWI/SNF subunit genes in different tissues. **(A)** RNA-Seq data for different tissues were extracted from a website (https://venanciogroup.uenf.br/cgi-bin/gmax_atlas/index.cgi). The color scale is shown at the right side of **(A)**. **(B)** Expression levels of twelve representative SWI/SNF subunits measured in soybean shoots, leaves, flowers, and roots using qRT-PCR. RMZ, root meristematic zone.

To analyze the expression patterns of the SWI/SNF components in soybean, we used publicly available transcriptome data from the Soybean Expression Atlas (Machado et al., 2020), including data for seven different tissues (root, cotyledon, shoot, flower, seed, leaf, and pod) and three root parts (differentiation, elongation, and meristematic zones). As shown in **Figure 4A**, the ATPase-coding genes *GmSYDs* and *GmBRMs* were expressed in nearly all the tested tissues, with the highest expression levels in flowers. *GmLFRs*, *GmARP4s*, *GmSNF5*, *GmSWI3As*, *GmSWI3Bs*, *GmSWI3Cs*, *GmSWI3Ds*, and *GmBRIPs* were also expressed in most of the tested tissues; however, the expression levels of some of the genes were low in seeds. Additionally, *GmARP7D* expression is highest in the cotyledons; *GmARP7A* and *GmBRD1* showed extremely low expression levels in almost all tissues. Finally, most of the SWI/SNF subunits were expressed in roots, especially in the root meristematic zone, including *GmMINU3*, *GmLFRs*, *GmARP4s*, *GmARP7B*, *GmSNF5*, *GmSWP73A*, and *GmSWI3s*. These results suggest that the genes encoding SWI/SNF subunits have specific spatiotemporal expression patterns during soybean growth and development.

We next confirmed the expression of some of these genes in roots, leaves, flowers, and shoots using qRT-PCR. Consistent with publicly available RNA-Seq data, *GmSYD1*, *GmLFR1*, *GmMINU3*, *GmSWI3A2*, and *GmSWI3Cs* were expressed in different tissues and organs, with the highest expression levels observed in flowers. The expression of *GmARP7A* was low compared to that of the other genes in all tissues. *GmMINU3* showed higher expression than *GmMINU1* (**Figure 4B**). However, the expression patterns of a few genes differed from those found using publicly available RNA-Seq data. For example, compared with *GmSYD1*, *GmSYD2* had lower expression levels in all tissues and *GmLFR2* was barely expressed in the tissues tested (**Figure 4B**). This discrepancy could result from the difference in soybean accessions (PI-567690 for the RNA-seq data; Williams 82 for our qRT-PCR results), the developmental stages, and the growth conditions in two separate assays.

### Expression analysis of SWI/SNF components genes under drought stress

**Figure 5:**
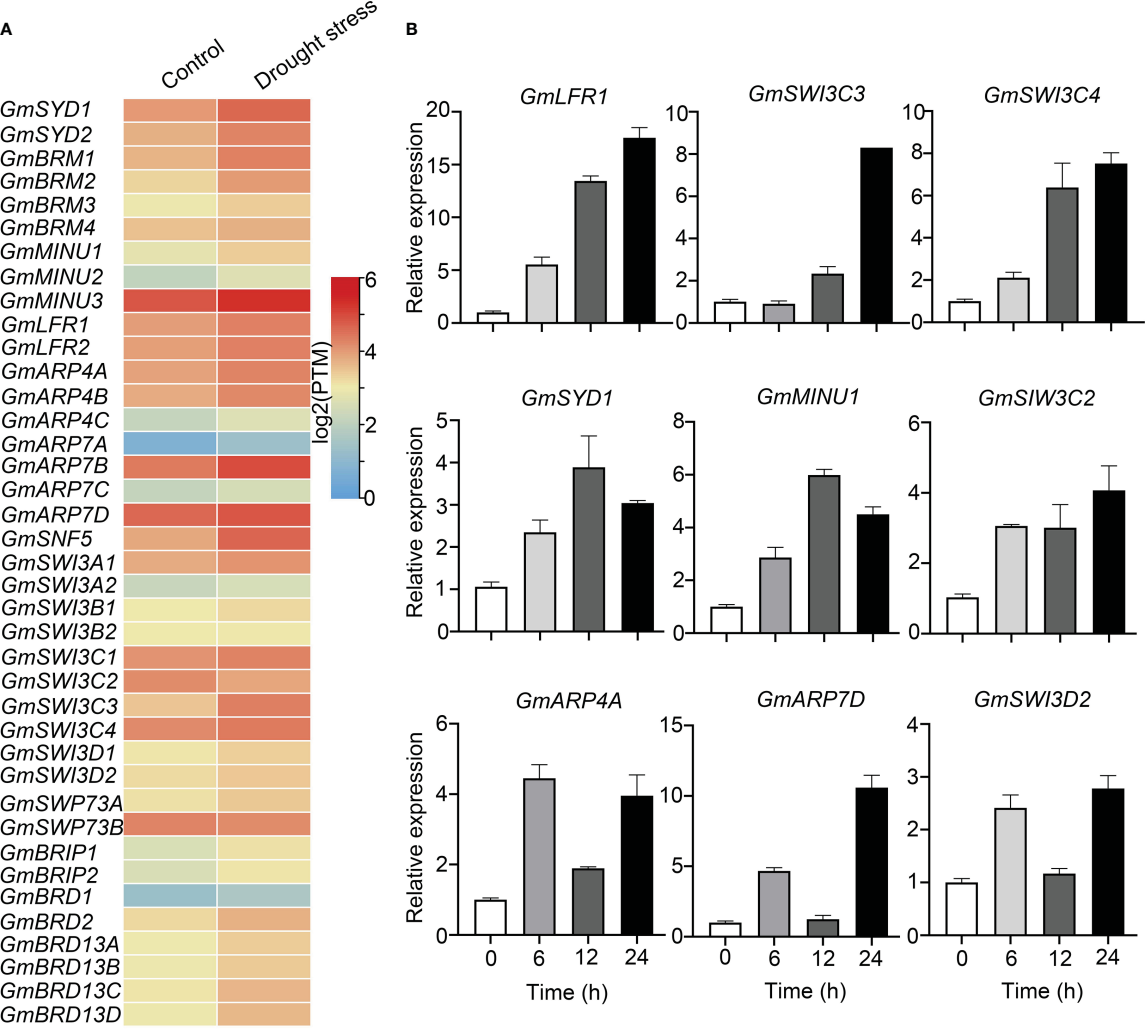
Transcriptional expression levels of soybean SWI/SNF subunit genes under drought-stress conditions. **(A)** RNA-Seq data from different tissues with or without drought stress treatment were acquired from a website (https://venanciogroup.uenf.br/cgi-bin/gmax_atlas/index.cgi). The color scale is shown at the right side of **(A)**. **(B)** The expression levels of nine SWI/SNF subunit genes were measured using qRT-PCR following 8% PEG-6000 treatment for 0, 6, 12, and 24 h.

Based on publicly available RNA-Seq data from plants treated with and without drought stress, we found that most genes encoding soybean SWI/SNF subunits were up-regulated (fold change ≥ 2) after drought treatment, including *GmBRMs*, *GmSYDs*, *GmMINU1*, *GmSNF5*, *GmSWI3C3, GmARP4B*, *GmARP7B*, *GmARP7D*, *GmLFRs*, and *GmBRIDs* (**Figure 5A**).

Next, we confirmed the transcriptional levels of several soybean SWI/SNF subunit genes by qRT-PCR after PEG treatment to imitate drought stress. The expression of *GmLFR1*, *GmSWI3C3*, and *GmSWI3C4* rose as the PEG treatment time increased. The expression of *GmARP4A*, *GmARP7D*, and *GmSWI3D2* also increased, but it fluctuated with treatment time (**Figure 5B**). The expression of *GmSYD1* and *GmMINU1* was induced most obviously after 12 h of treatment (**Figure 5B**). Thus, all the selected genes (*GmSYD1*, *GmMINU1*, *GmLFR1*, *GmARP4A*, *GmARP7D*, *GmSWI3C2*, *GmSWI3C3*, *GmSWI3C4*, and *GmSWI3D2*) showed increased expression following PEG-6000 treatment, but with different time courses. Together with our finding that the promoters of most SWI/SNF subunit genes harbored stress-responsive elements, these results indicate that soybean SWI/SNF subunit genes may be involved in drought stress responses. Notably, *GmLFR1* was induced significantly by PEG treatment, and the expression of its paralog *GmLFR2* was not detectable in the presence or absence of drought stress (**Figures 4B**, **S2**). Thus, *GmLFR1* was selected for further analysis.

### *GmLFR1* overexpression in hairy roots negatively regulates drought tolerance

**Figure 6:**
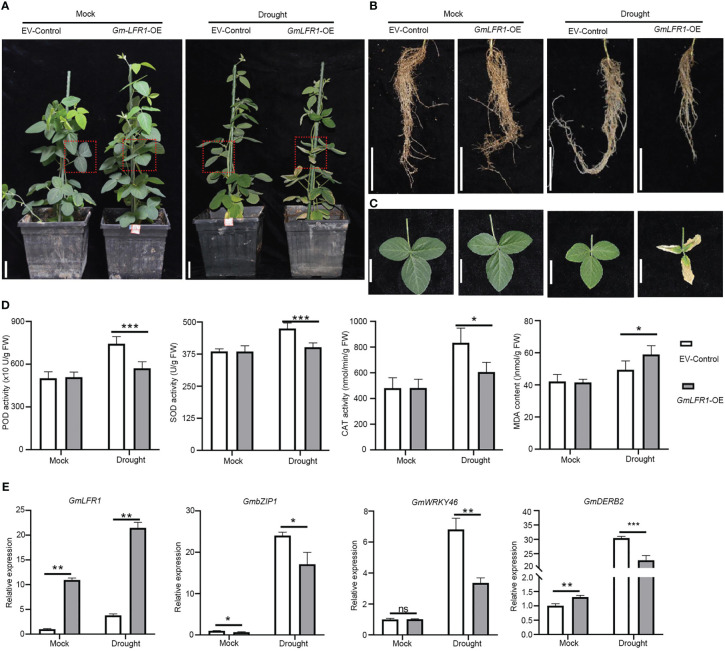
Functional analysis of *GmLFR1*-OE and EV-control plants with transgenic soybean hairy roots under mock and PEG conditions. **(A)** The phenotypes of *GmLFR1*-OE and EV-control transgenic plants under mock and PEG conditions. Leaves at similar positions from different plants (indicated by red boxes) are enlarged in **(B)**. The leaves **(B)** and roots **(C)** of *GmLFR1*-OE and EV-control transgenic plants under mock and PEG conditions. **(D)** POD, SOD, and CAT activity levels and the MDA contents of *GmLFR1*-OE and EV-control transgenic plants under mock and PEG conditions. **(E)** Expression levels based on qRT-PCR analyses of several stress-responsive genes in *GmLFR1*-OE and EV-control transgenic plants under mock and PEG conditions. Bars indicate means ± SD of three biological replicates. *(*p* < 0.05), ** (*p* < 0.01), and *** (*p* < 0.001) indicate significant differences as determined by Student’s *t*-tests. Bar = 5 cm.

To explore whether *GmLFR1* is involved in the drought response of soybean plants, we generated *GmLFR1-*OE and empty vector control (EV-control) transgenic plants using *A. rhizogenes*-mediated hairy root transformation. The *GmLFR1*-OE plants had similar aboveground and root appearances as the EV-control plants under mock conditions (**Figures 6A, B**). However, *GmLFR1*-OE plants treated with 10% PEG-6000 showed significantly more severe leaf curling and wilting and shorter roots compared to the EV-controls (**Figures 6B, C**). Also, the peroxidase (POD), catalase (CAT), and superoxide dismutase (SOD) activity levels in the *GmLFR1*-OE plants were significantly lower than those in the EV-control plants, but the MDA content was significantly higher than that in the EV-control plants under drought stress conditions (**Figure 6D**). However, there was no significant difference in these physiological and biochemical parameters between the *GmLFR1*-OE and EV-control plants under normal growth conditions. Thus, *GmLFR1* overexpression negatively regulates drought tolerance in soybean.

To clarify the possible regulatory mechanisms whereby the overexpression of *GmLFR1* affects drought tolerance in soybean, we examined the transcriptional expression patterns of drought stress-responsive genes (*GmDREB2*, *GmbZIP1*, and *GmWRKY46*) in *GmLFR1*-OE and EV-control hairy roots with or without PEG-6000 treatment using qRT-PCR. The expression level of *GmLFR1* in the transgenic hairy roots was significantly higher than that in the EV-controls, indicating successful overexpression (**Figure 6E**). The transcript levels of *GmDREB2, GmbZIP1,* and *GmWRKY46* in GmLFR1-OE plants were significantly lower than those in EV-control after drought treatment (**Figure 6E**). The misregulation of these genes is consistent with the drought-sensitive defect of the *GmLFR1*-OE plants. These results show that *GmLFR1* may regulate the transcription of drought-responsive genes in soybean to mediate the response to dehydration stress. Our results also show that under normal growth conditions, the transcript level of *GmDREB2* was upregulated while that of *GmbZIP1* was down-regulated in *GmLFR1*-OE plants compared to EV-control plants, suggesting that *GmLFR1* may also regulate the expression of these genes under normal growth conditions.

### *GmLFR1* regulates drought stress tolerance in Arabidopsis

**Figure 7:**
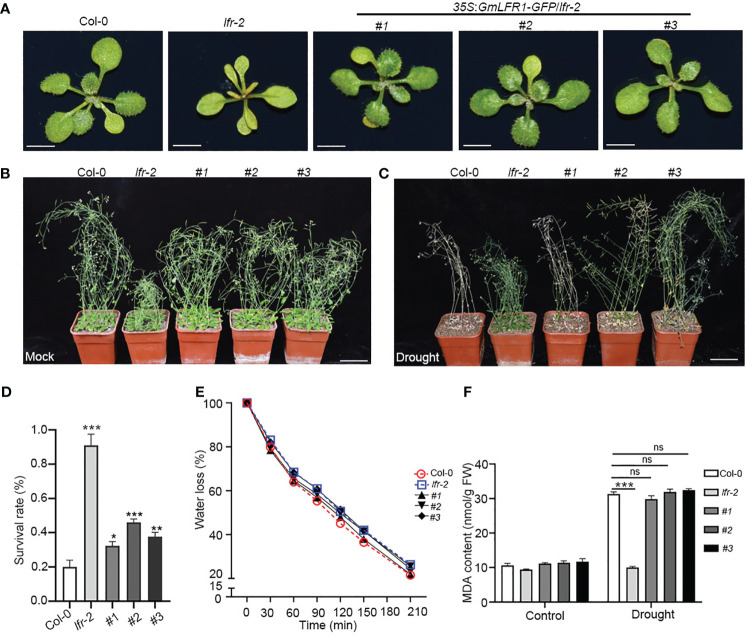
Phenotypes of WT Col-0, *lfr-2*, and transgenic Arabidopsis plants under normal and drought stress conditions. **(A)** The overall morphologies of 15-day-old WT Col-0, *lfr-2*, and *35S*:*GmLFR1-GFP/lfr-2* transgenic plants grown under long-day conditions. Bars = 1 cm. **(B, C)** The growth states of WT Col-0, *lfr-2*, and *35S*:*GmLFR1-GFP/lfr-2* transgenic plants grown in soil for 3 weeks without **(B)** or with drought treatment **(C)**. Bars = 5 cm. **(D)** Survival rate of 6-week-old WT Col-0, *lfr-2*, and three *35S*:*GmLFR1-GFP/lfr-2* transgenic lines after re-watering for 5 days. Values are the mean ± SE from two independent experiments (n > 53). **(E)** Relative water loss in WT Col-0, *lfr-2*, and three *35S*:*GmLFR1-GFP/lfr-2* transgenic lines. **(F)** MDA contents of WT Col-0, *lfr-2*, and three *35S*:*GmLFR1-GFP/lfr-2* transgenic plants treated with or without drought stress. Statistically significant differences are indicated by asterisks (*P<0.05, **P < 0.01, and ***P < 0.001; Student’s t-test).

To further explore whether the functions of *GmLFR1* and *AtLFR* are conserved, we generated GmLFR1-GFP driven by the CaMV 35S promoter (*35S*:*GmLFR1-GFP*) in the background of the heterozygous Arabidopsis *LFR* mutant (*lfr-2*/*+*) because the homozygous mutant is sterile. In the T3 generation, we analyzed three independent homozygous transgenic Arabidopsis lines of *35S*:*GmLFR1-GFP*/*lfr-2* (#1, #2, and #3). We found that heterologous expression of *GmLFR1* successfully restored the leaf developmental defects seen in *lfr-2* (**Figures 7A**, **S3**). This result indicates that the molecular functions of *GmLFR1* and *AtLFR* are conserved under normal conditions.

We next explored whether *AtLFR* is also involved in drought stress. Three-weeks drought stress was applied to 3-week-old wild-type (WT) Col-0 and *lfr-2* mutant plants. The *lfr-2* mutants showed an obvious drought-tolerant phenotype (**Figures 7B, C**). Following 5 days of recovery, more than 92.85% of the *lfr-2* plants survived, compared with 20% of the WT plants (**Figure 7D**). Consistently, the water loss rates of *lfr-2* were slower than those of WT Col-0 plants (**Figure 7E**), and the leaf MDA content in Col-0 was increased significantly, while there was no significant difference in *lfr-2*, after drought treatment (**Figure 7F**). These data indicate that *AtLFR* may play a negative regulatory role in drought tolerance in Arabidopsis. Intriguingly, the enhanced drought tolerance of *lfr-2* could be substantially rescued by the overexpression of *GmLFR1* (**Figures 7B–F**). Taken together, these results suggest that *GmLFR1* and *AtLFR* are functionally conserved in plant drought response.

### GmLFR1 localizes to the nucleus

**Figure 8:**
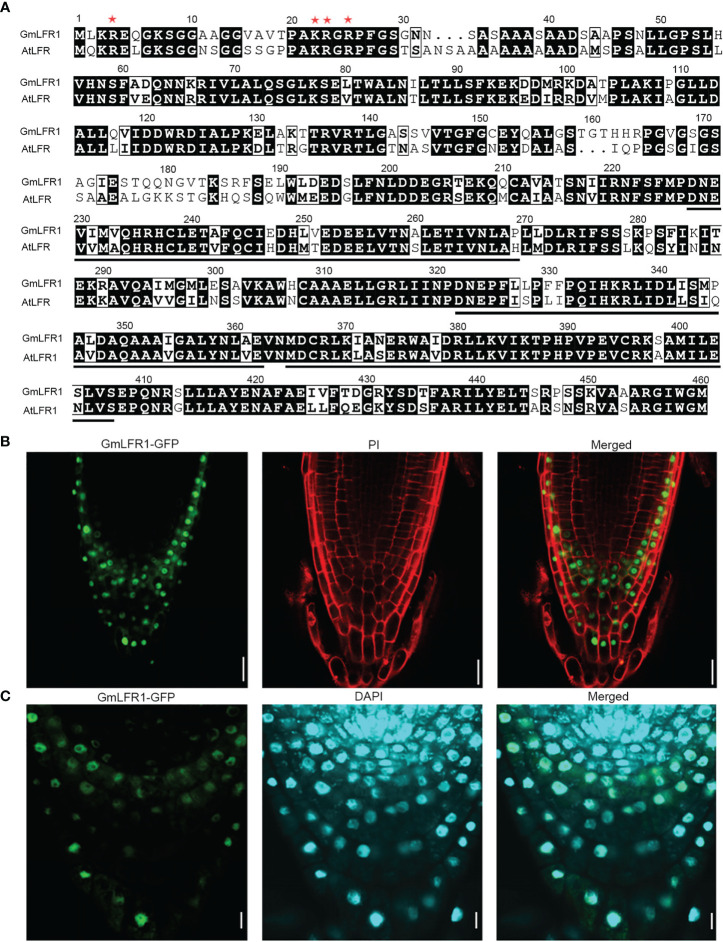
Subcellular localization of GmLFR1. **(A)** Sequence alignment of the conserved amino acid residues in GmLFR1 and AtLFR. The ARM domains are underlined. Red asterisks indicate the conserved amino acid residues that are essential for nuclear localization. **(B, C)** GFP fluorescence in *35S*:*GmLFR1-GFP/lfr-2* roots; PI staining indicates cell membranes **(B)** and DAPI staining indicates nuclei **(C)**. Bars = 10 μm.

SWI/SNF subunits generally function in the nucleus (Wagner and Meyerowitz, 2002; Farrona et al., 2004; Sarnowski et al., 2005). We previously showed that AtLFR localizes to the nucleus and that the N-terminal amino acids (1–25) of AtLFR, including the basic amino acids lysine at position 22 (K22) and arginine at positions 4, 23, and 25 (R4, R23, and R25), are essential for its nuclear localization (Yuan et al., 2012). To gain insight into the subcellular localization of GmLFR1, a protein sequence alignment was conducted between GmLFR1 and AtLFR1. The amino acid residues essential for nuclear localization were conserved in GmLFR1 (**Figure 8A**), suggesting that GmLFR1 is also a nuclear protein. To confirm this, we analyzed the levels of GFP florescence in the roots of the *35S*:*GmLFR1-GFP*/*lfr-2* transgenic lines using confocal microscopy and found that GmLFR, like its Arabidopsis ortholog AtLFR, is a nucleus-localized protein (**Figures 8B, C**). This subcellular localization pattern is consistent with its role as a SWI/SNF subunit.

## Discussion

SWI/SNF complex regulate the conformation of chromatin and affect DNA accessibility using energy generated by ATP hydrolysis, which is an important mechanism for the transcriptional regulation of eukaryotic gene expression (Clapier and Cairns, 2009; Narlikar et al., 2013; Clapier et al., 2017). SWI/SNF complex are composed of several evolutionarily conserved epigenetic regulators, including an ATPase subunit and multiple other components, and are widely involved in plant growth, development, and stress responses (Abrams et al., 1986; Han and Wagner, 2014; Sarnowska et al., 2016). SWI/SNF complex was first identified in yeast, and to data, more than 19 SWI/SNF complex components have been identified in Arabidopsis, and some components have been identified and analyzed in rice (OsLFR and OsSWI3C) and maize (ZmCHC101) (Yu et al., 2016; Yu et al., 2018; Qi et al., 2020; Yang et al., 2020). In this study, we used the amino acid sequences of 19 SWI/SNF components from Arabidopsis as references to systematically search for SWI/SNF subunit genes in a soybean database. We identified 39 SWI/SNF component genes in soybean; this is almost twice as many as in the diploid model plant Arabidopsis, and most Arabidopsis SWI/SNF component genes had two to four paralogs in soybean (**Table 1**). Soybean is a paleopolyploid plant that has undergone two genome duplication events during its long evolutionary history, yielding multiple copies of nearly 75% of its genes (Schmutz et al., 2010). The increased number and diversity of SWI/SNF subunit genes in soybean are consistent with its paleopolyploid status. The increased number of soybean SWI/SNF subunit genes implies that there may be a more diverse group of SWI/SNF complex with more complex and fine-tuned regulatory mechanisms in soybean than in Arabidopsis. The existence of paralogs of most SWI/SNF subunit genes suggests that there is functional redundancy between them; our gene expression pattern analysis revealed differences among some of the paralogs, which could indicate divergent functions. These speculations must be confirmed in future studies. Additionally, the exact composition of soybean SWI/SNF complex should be analyzed using proteomic methods such as immunoprecipitation combined with mass spectrometry (IP-MS).

Arabidopsis SWI/SNF subunits are involved in a variety of biotic and abiotic stress responses (Han et al., 2012; Song et al., 2021). In this study, we identified multiple *cis*-regulatory elements related to ABA and abiotic stresses such as drought by performing an *in silico cis*-regulatory element analysis of the putative promoter regions of soybean SWI/SNF subunit genes. Transcriptional expression analyses using qRT-PCR combined with publicly available gene expression profiling data demonstrated that about 25 SWI/SNF subunit genes were induced by drought stress. Stress phenotype identification demonstrated that *GmLFR1*-OE plants exhibited significantly accelerated leaf dehydration, shorter roots, lower CAT, SOD, and POD activity levels, and higher MDA contents than the EV-controls after PEG treatment (**Figure 6**). Thus, these results indicate that the overexpression of *GmLFR1* reduced the resistance of plants to drought. It was previously reported that the overexpression of SWI/SNF complex subunit OsSWI3C leads to decreased drought resistance in rice (Yang et al., 2020), and BRM negatively regulates drought tolerance in Arabidopsis (Han et al., 2012). Together, these results may indicate that at least some of the SWI/SNF subunit genes are negative regulators of drought stress.

The plants have evolved sophisticated regulatory networks that can fine-tune the balance between stress resistance and growth in a challenging environment (Zhou et al., 2018). Under adverse conditions, the plants must induce some positive stress-responsive genes (*e.g*., *DREB*s in abiotic stress) to improve plant fitness (Lata and Prasad, 2011). However, over-stimulation of these genes may be detrimental to plant growth (Zhao et al., 2017). Therefore, even under stressful conditions, plants may also need to induce some genes to avoid over-response to the environment and keep the plant growth capacity. For example, the transcription of *Patellin1* (*PATL1*), a negative regulator of salt tolerance, is induced by salt, which may be a negative feedback mechanism in the regulation of plant salt tolerance (Zhou et al., 2018). *AtCaM4* is cold-induced and negatively regulates freezing tolerance in Arabidopsis (Chu et al., 2018). On the contrary, some positive stress-tolerance regulators may be repressed by the stress. For example, the Arabidopsis ICE1 protein declines after cold treatment, which could be of benefit to plants to balance cold response and growth (Zhao et al., 2017). Our results showed that a subset of SWI/SNF genes were induced by drought stress, and we demonstrated that *GmLFR1* is a negative regulator of drought tolerance (**Figure 6**). We also found that the enhanced drought tolerance of *lfr-2* could be partially rescued by the overexpression of *GmLFR1* (**Figure 7**), suggesting that the functions of *GmLFR1* and *AtLFR* are conserved and they both play a negative regulatory role in plant drought tolerance. Moreover, we found that the transcript levels of *GmDREB2*, *GmbZIP1*, and *GmWRKY46* in *GmLFR1*-OE plants were significantly lower than that in the EV-controls after PEG-6000 treatment (**Figure 6E**), which is consistent with the previous reports showing that the Arabidopsis SWI/SNF core ATPase *BRM* are required for the restricting stress response gene expression (*e.g*., *ABA INSENSITIVE5*) both under normal growth and ABA treatment conditions. Therefore, at least some of the soybean SWI/SNF components (*e.g*., GmLFR1) may be involved in the negative feedback regulation mechanism of stress response gene expression to fine-tune plant growth and stress tolerance, which is interesting to be tested in the future.

In addition, SWI/SNF subunits are involved in growth and development in the roots, flowers, and leaves of Arabidopsis (Farrona et al., 2004; Bezhani et al., 2007; Lin et al., 2018). By analyzing the expression patterns of soybean SWI/SNF subunit genes, we found that most genes were expressed in soybean roots, leaves, and flowers (**Figure 4**). We also identified multiple *cis*-regulatory elements related to hormone signaling in the putative promoter regions of soybean SWI/SNF subunit genes. Thus, it will be of interesting to decipher whether the SWI/SNF subunits function in soybean development using loss-of-function mutants and overexpression lines in the future.

## Conclusion

We identified 39 SWI/SNF subunit genes from the soybean genome and characterized their structures and chromosome locations. We also constructed phylogenetic trees and analyzed the properties of their encoded proteins. *Cis*-regulatory element and transcriptional expression analyses indicated that SWI/SNF complex may play important roles in the response of soybean to environmental stress. Analyses of phenotype, physiological indicators, and stress-responsive gene expression showed that *GmLFR1*, which is conserved in Arabidopsis, plays a negative role in drought tolerance in soybean. Our study provides insight into the composition of SWI/SNF complex in soybean and a basis for further research into their roles in abiotic stress responses.

## Data availability statement

The datasets presented in this study can be found in online repositories. The names of the repository/repositories and accession number(s) can be found in the article/**Supplementary Material**.

## Author contributions

HTZ and CY designed the research and proposed the research proceeding. QC, XS, LA, HWZ, XT, JT and QW performed the experiments; QC, HTZ, SC and MZ analyzed the data; QC, HTZ wrote, reviewed and edited the manuscript. All authors contributed to the article and approved the submitted version.
