# Supplementary material for: Genome-wide identification of genes encoding SWI/SNF components in soybean and the functional characterization of GmLFR1 in drought-stressed plants
Source: Front Plant Sci. 2023 May 15;14:1176376. doi: 10.3389/fpls.2023.1176376 (PMC10225534; doi:10.3389/fpls.2023.1176376)
Supplement: Supplementary file 1 [file DataSheet_1.docx]

**
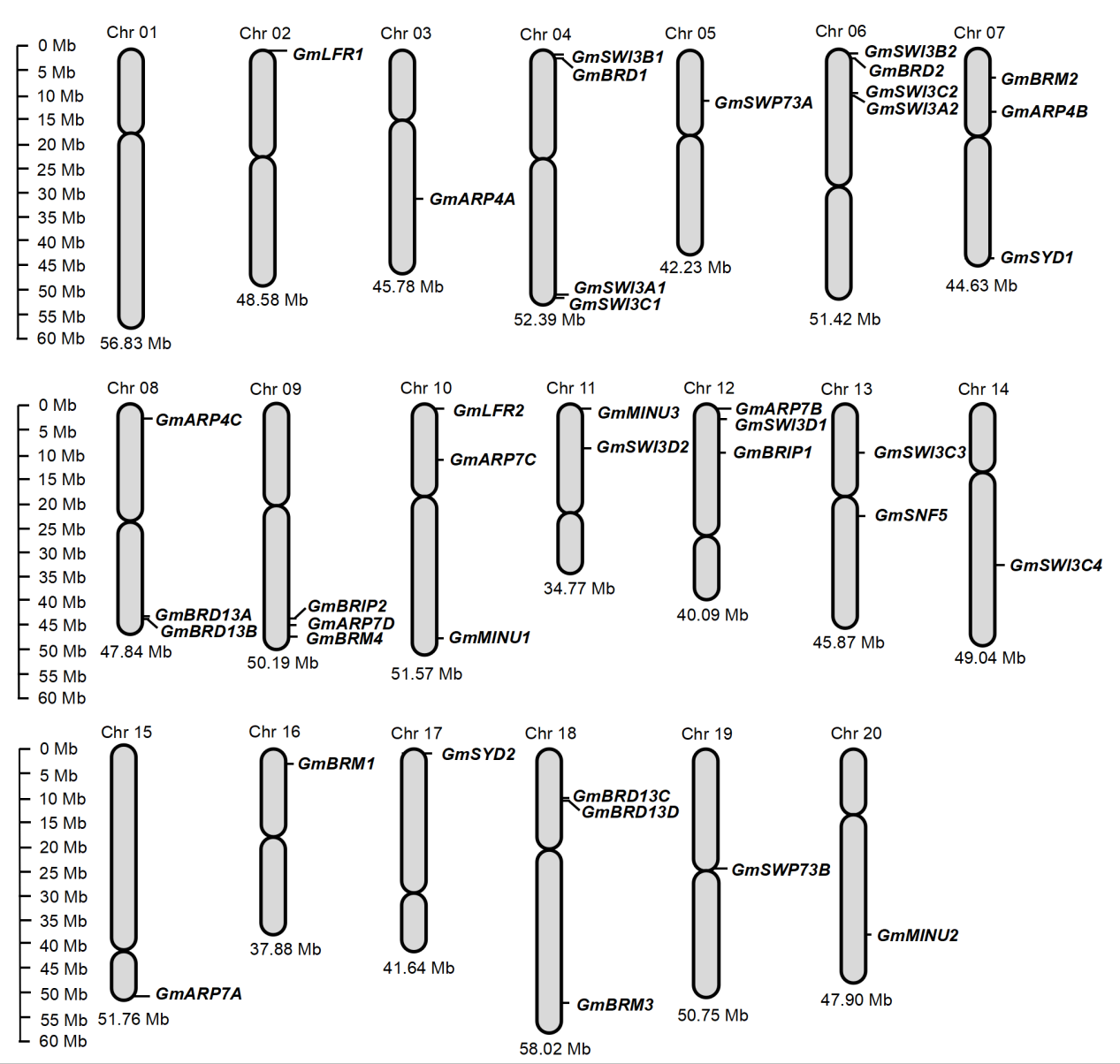
**

**Supplementary Fig. 1.** Chromosomal distribution of the SWI/SNF subunit genes in soybean. The soybean genome encodes 39 SWI/SNF complex subunits, which are mapped to different chromosomes as shown in the figure with black lines indicating their position. The scale bars on the left and the numbers below each chromosome indicate the sizes of the chromosomes.

**
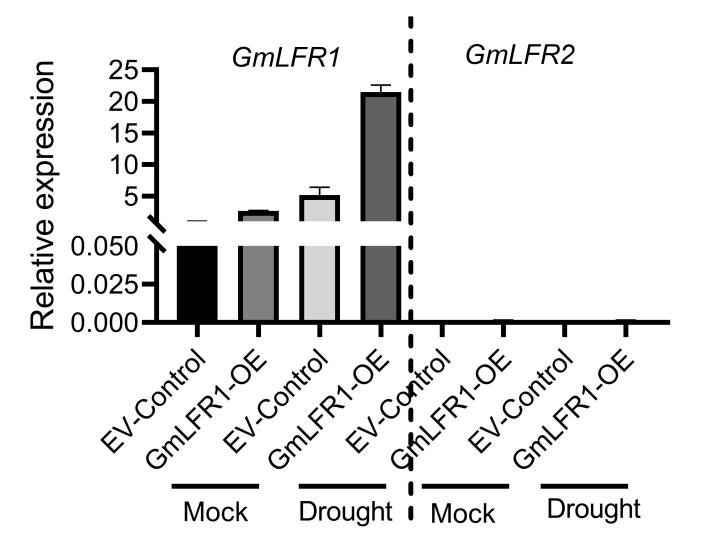
**

**Supplementary Fig. 2.** Expression levels based on qRT-PCR analyses of *GmLFR1* and *GmLFR2* genes in *GmLFR1*-OE and EV-control transgenic plants under mock and drought conditions.

**
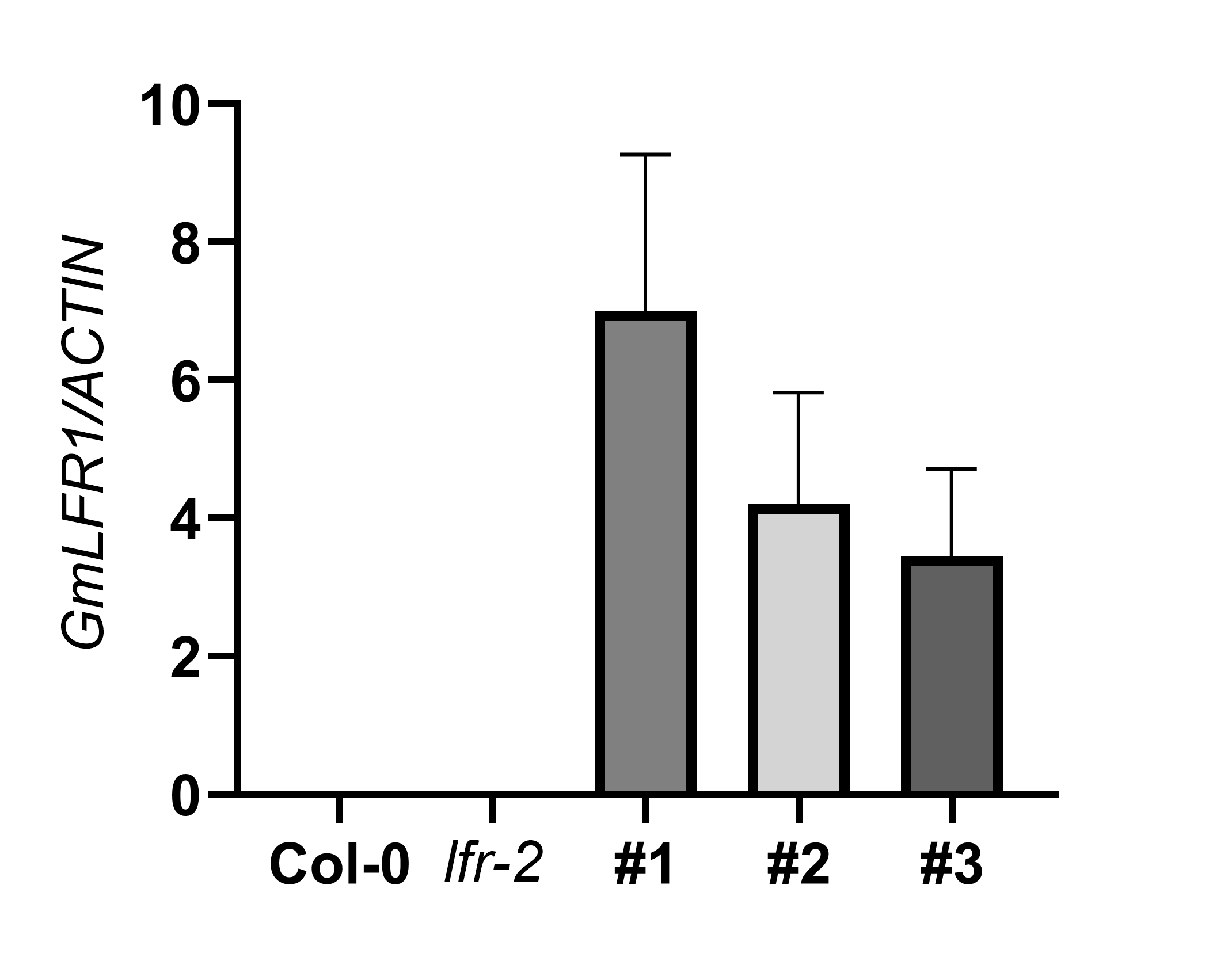
**

**Supplementary Fig. 3.** The RNA level analysis of GmLFR1 in Col-0, *lfr-2* and *35S:GmLFR1-GFP/lfr-2* transgenic plants.

| **Supplementary Table 1. TPM values for soybean SWI/SNF subunit genes in different tissues.** |
| --- |

| Gene Name | Cotyled-on | Leaves | Flower | Pod | Seed | Shoot | Root | Root meristem-atic zone | Root elongation zone | Root differentiati-on zone |
| --- | --- | --- | --- | --- | --- | --- | --- | --- | --- | --- |
| GmSYD1 | 16.91 | 17.21 | 29.74 | 15.34 | 4.51 | 14.94 | 13.75 | 17.27 | 11.63 | 16.80 |
| GmSYD2 | 14.32 | 15.42 | 25.65 | 13.64 | 4.53 | 13.43 | 14.15 | 19.58 | 13.97 | 17.61 |
| GmBRM1 | 20.16 | 15.63 | 22.77 | 11.80 | 4.70 | 10.61 | 10.95 | 14.68 | 8.78 | 8.84 |
| GmBRM2 | 10.47 | 11.60 | 14.69 | 7.46 | 3.50 | 6.81 | 9.14 | 8.31 | 6.72 | 8.69 |
| GmBRM3 | 9.68 | 11.15 | 16.10 | 9.98 | 5.84 | 9.31 | 10.93 | 8.54 | 8.91 | 11.08 |
| GmBRM4 | 16.93 | 15.81 | 22.89 | 14.02 | 6.34 | 14.03 | 17.63 | 16.69 | 14.90 | 16.71 |
| GmMINU1 | 4.68 | 6.18 | 7.22 | 4.37 | 1.68 | 5.41 | 5.97 | 8.80 | 6.07 | 5.16 |
| GmMINU2 | 2.18 | 4.75 | 5.44 | 4.57 | 1.83 | 3.16 | 7.59 | 9.03 | 5.77 | 4.51 |
| GmMINU3 | 26.23 | 25.05 | 30.07 | 19.54 | 10.30 | 21.86 | 20.97 | 33.04 | 27.26 | 30.14 |
| GmLFR1 | 11.60 | 14.95 | 13.63 | 11.39 | 7.84 | 9.36 | 10.60 | 24.45 | 16.02 | 15.05 |
| GmLFR2 | 10.93 | 13.15 | 15.33 | 11.65 | 8.75 | 8.16 | 11.34 | 19.89 | 16.07 | 14.15 |
| GmARP4A | 7.03 | 17.09 | 14.43 | 13.33 | 6.38 | 7.21 | 11.47 | 29.06 | 16.73 | 15.77 |
| GmARP4B | 7.34 | 22.21 | 19.61 | 16.93 | 7.76 | 9.97 | 14.09 | 39.86 | 18.56 | 13.71 |
| GmARP4C | 2.75 | 8.78 | 10.02 | 10.01 | 7.06 | 6.06 | 10.92 | 30.66 | 16.95 | 12.46 |
| GmARP7A | 0.00 | 4.01 | 2.94 | 2.82 | 2.10 | 1.20 | 2.91 | 11.73 | 4.98 | 3.39 |
| GmARP7B | 10.74 | 16.65 | 22.45 | 20.07 | 16.07 | 9.14 | 13.17 | 34.54 | 19.28 | 21.75 |
| GmARP7C | 1.78 | 7.97 | 6.91 | 4.37 | 2.92 | 1.80 | 6.99 | 15.47 | 5.32 | 4.21 |
| GmARP7D | 50.31 | 23.06 | 20.96 | 18.58 | 10.80 | 16.07 | 9.10 | 11.26 | 10.11 | 13.37 |
| GmSNF5 | 13.19 | 17.74 | 17.18 | 14.74 | 9.87 | 11.93 | 13.38 | 39.66 | 25.65 | 19.03 |
| GmSWI3A1 | 10.58 | 14.26 | 11.57 | 11.43 | 5.72 | 9.82 | 11.03 | 24.22 | 14.36 | 11.13 |
| GmSWI3A2 | 2.69 | 5.39 | 4.66 | 3.34 | 2.31 | 2.26 | 4.32 | 11.31 | 4.67 | 4.81 |
| GmSWI3B1 | 12.48 | 12.73 | 11.69 | 9.72 | 5.85 | 8.85 | 13.09 | 15.43 | 10.85 | 9.31 |
| GmSWI3B2 | 4.10 | 6.88 | 8.08 | 7.92 | 5.83 | 5.71 | 8.20 | 20.71 | 11.70 | 10.35 |
| GmSWI3C1 | 10.13 | 17.00 | 24.62 | 16.46 | 7.40 | 9.61 | 9.78 | 15.94 | 10.51 | 10.60 |
| GmSWI3C2 | 13.13 | 20.73 | 19.26 | 14.82 | 7.16 | 13.68 | 11.46 | 15.75 | 12.84 | 11.89 |
| GmSWI3C3 | 17.30 | 14.02 | 22.77 | 14.02 | 8.77 | 12.08 | 22.38 | 12.06 | 13.62 | 16.08 |
| GmSWI3C4 | 14.34 | 16.59 | 18.76 | 13.67 | 6.20 | 10.23 | 10.47 | 15.13 | 11.18 | 12.45 |
| GmSWI3D1 | 5.06 | 7.56 | 10.41 | 6.58 | 3.14 | 5.63 | 8.65 | 15.25 | 10.96 | 10.07 |
| GmSWI3D2 | 6.36 | 8.08 | 11.30 | 6.37 | 2.13 | 6.55 | 9.02 | 12.96 | 10.09 | 9.53 |
| GmSWP73A | 12.91 | 11.69 | 14.48 | 11.61 | 3.63 | 9.70 | 14.87 | 19.31 | 14.98 | 15.71 |
| GmSWP73B | 8.21 | 10.62 | 9.99 | 8.72 | 2.33 | 8.84 | 8.31 | 17.34 | 12.07 | 11.80 |
| GmBRIP1 | 9.49 | 11.03 | 13.80 | 9.07 | 7.68 | 6.39 | 6.94 | 8.81 | 6.34 | 6.48 |
| GmBRIP2 | 4.97 | 9.58 | 11.85 | 7.94 | 6.06 | 7.19 | 7.90 | 7.74 | 5.79 | 6.52 |
| GmBRD1 | 1.36 | 2.24 | 6.27 | 2.32 | 1.25 | 1.43 | 6.26 | 5.04 | 4.43 | 4.07 |
| GmBRD2 | 6.84 | 6.85 | 9.22 | 6.71 | 3.08 | 3.12 | 5.18 | 7.23 | 5.61 | 4.93 |
| GmBRD13A | 11.78 | 10.58 | 19.22 | 11.53 | 4.86 | 9.09 | 11.57 | 10.10 | 8.07 | 11.88 |
| GmBRD13B | 11.67 | 9.41 | 17.01 | 8.99 | 3.56 | 8.45 | 10.10 | 8.55 | 8.05 | 10.12 |
| GmBRD13C | 20.80 | 13.09 | 23.98 | 17.54 | 7.15 | 12.90 | 12.85 | 10.86 | 11.27 | 13.87 |
| GmBRD13D | 14.42 | 9.65 | 12.17 | 9.53 | 3.49 | 10.07 | 10.11 | 8.24 | 10.96 | 11.87 |

**Supplementary Table 2. TPM values for soybean SWI/SNF subunit genes under drought treatment.**

| Gene Name | Control condition | Drought stressed for 15 days |
| --- | --- | --- |
| GmSYD1 | 15.2811 | 23.8315 |
| GmSYD2 | 12.4851 | 18.2577 |
| GmBRM1 | 12.0032 | 18.9825 |
| GmBRM2 | 8.8898 | 15.1245 |
| GmBRM3 | 6.6571 | 9.6154 |
| GmBRM4 | 10.6538 | 12.4059 |
| GmMINU1 | 5.9440 | 9.6685 |
| GmMINU2 | 3.2019 | 5.3697 |
| GmMINU3 | 28.0698 | 39.8486 |
| GmLFR1 | 14.8021 | 19.1176 |
| GmLFR2 | 14.5854 | 19.1513 |
| GmARP4A | 14.0306 | 18.3133 |
| GmARP4B | 13.0012 | 17.6909 |
| GmARP4C | 3.4553 | 5.3232 |
| GmARP7A | 0.6543 | 1.4924 |
| GmARP7B | 20.2647 | 30.8942 |
| GmARP7C | 3.4030 | 4.5838 |
| GmARP7D | 24.0417 | 27.7840 |
| GmSNF5 | 13.3053 | 24.6480 |
| GmSWI3A1 | 13.1737 | 16.0717 |
| GmSWI3A2 | 3.6760 | 4.7722 |
| GmSWI3B1 | 7.1057 | 8.6043 |
| GmSWI3B2 | 7.1793 | 7.3336 |
| GmSWI3C1 | 16.2378 | 18.4954 |
| GmSWI3C2 | 17.0763 | 13.7575 |
| GmSWI3C3 | 10.1267 | 19.6392 |
| GmSWI3C4 | 17.5021 | 20.2367 |
| GmSWI3D1 | 7.4518 | 9.3764 |
| GmSWI3D2 | 8.4791 | 10.0196 |
| GmSWP73A | 7.8320 | 9.9203 |
| GmSWP73B | 18.6643 | 17.0646 |
| GmBRIP1 | 4.8687 | 7.8776 |
| GmBRIP2 | 5.0095 | 7.5548 |
| GmBRD1 | 1.4619 | 2.1452 |
| GmBRD2 | 8.6307 | 12.3899 |
| GmBRD13A | 7.0038 | 9.5937 |
| GmBRD13B | 6.6656 | 9.8663 |
| GmBRD13C | 7.6141 | 11.8641 |
| GmBRD13D | 6.9763 | 11.6121 |

| **Supplementary Table 3. List of primers used in this study.** | |  |
| --- | --- | --- |
| Primer Name | Primer sequences(5'-3') | Purpose |
| GmARP4A-Q-FP | GCTGGTTATGCTGGTGAAGA | For qRT-PCR |
| GmARP4A-Q-RP | GGTCTCTACGGTACCCCAAGG |  |
| GmARP4B-Q-FP | GAAGATGCTCCCAAGGCTGTG |  |
| GmARP4B-Q-RP | CCAAGGACTGGGATCCTACATACAA |  |
| GmARP4C-Q-FP | ACAGAAAGCTACAAACTCTACTC |  |
| GmARP4C-Q-RP | TCTGCAAAACTCTCCATACCAGGG |  |
| GmARP7A-Q-FP | AACTTGACATTCGATGACATTGCTG |  |
| GmARP7A-Q-RP | ACCCTCAATCACTGGTGCAATAT |  |
| GmARP7B-Q-FP | TTCACTGGCTGACAATGTCACC |  |
| GmARP7B-Q-FP | GCCTGAGATACGTCCCACAG |  |
| GmARP7C-Q-FP | CCGGCAGTCGATGACGTTG |  |
| GmARP7C-Q-FP | ACCCTCAATCACTGGTGCAATAT |  |
| GmARP7D-Q-FP | ACTGATCCACTTTGTACCCCTAAGG |  |
| GmARP7D-Q-RP | GTGGATTGGACTTGCCAAGTTCTATT |  |
| GmBRIP1-Q-FP | AGCAACAACTTCTGCTACAACAACAG |  |
| GmBRIP1-Q-RP | GGGAGCGGGATTGGGGCTA |  |
| GmBRIP2-Q-FP | GCAGCAACAACAACAACAACAACA |  |
| GmBRIP2-Q-RP | GCGGGATTGAGGTTAGGGTTAGG |  |
| GmBRM1-Q-FP | ACTACAGGAGGAGATGACACGATAT |  |
| GmBRM1-Q-RP | TGGGTCGTCCCCTTTTTCTTTCA |  |
| GmBRM2-Q-FP | CTCCATGCACAACAAAGACCTACT |  |
| GmBRM2-Q-RP | GTGCTATTTGTTGTCCTTGCTCCA |  |
| GmBRM3-Q-FP | AGTTCAGGTGCAAGGCACAC |  |
| GmBRM3-Q-RP | ACTCATTTCTAACAGGAGTTCTGCC |  |
| GmBRM4-Q-FP | TTTGCAACCCACACCTCCTGT |  |
| GmBRM4-Q-RP | CTAACTCATTTCTGCCAATTCCATG |  |
| GmSNF5-Q-FP | ACTCCGATTTCGGGTTTCTACAGA |  |
| GmSNF5-Q-RP | GGAGGGAGCTTCAAGTCTTTAACAG |  |
| GmSWI3A1-Q-FP | GCGGATTCAGATTCGGAACTGGA |  |
| GmSWI3A1-Q-RP | CCTTCTGGAGGGCTCTTCTCTG |  |
| GmSWI3A2-Q-FP | CCGAATTCGTTGAAGCCTATGTTGT |  |
| GmSWI3A2-Q-RP | ATCCTGTGTACATCGGTAATGTCCA |  |
| GmSWI3B1-Q-FP | AACCTCTCCCTTCCACCACC |  |
| GmSWI3B1-Q-RP | CCACGAGAACCACCGAGAGT |  |
| GmSWI3B2-Q-FP | ACCGAACCTCTCCCTTCCTCC |  |
| GmSWI3B2-Q-RP | TGACGCACCTCGCATTCGTC |  |
| GmSWI3C1-Q-FP | CCGGTTCTCGAAAACGTGTCT |  |
| GmSWI3C1-Q-RP | AAGAAATGCGGCACCACTTGC |  |
| GmSWI3C2-Q-FP | CCGATCACGAAGAACGAGAAA |  |
| GmSWI3C2-Q-RP | TCCCACAGTTCCAAGACGTGC |  |
| GmSWI3C3-Q-FP | GTTGCTCAAATGGGGATTCTGCA |  |
| GmSWI3C3-Q-RP | CCCGGAATTATTCCTTGACAATGCA |  |
| GmSWI3C4-Q-FP | CTGGGAGCACGGCACATAT |  |
| GmSWI3C4-Q-RP | GGATAGGCCTGCTTTGGCA |  |
| GmSWI3D1-Q-FP | GGTTCCTCCTCCGCACCCT |  |
| GmSWI3D1-Q-RP | CCTTTTTGAGCTGCTCAGCCA |  |
| GmSWI3D2-Q-FP | ATTCACCTGGCACCGAGCT |  |
| GmSWI3D2-Q-RP | CGGCTGTCCCTTGACTTCTAAAGC |  |
| GmSWP73A-Q-FP | GTGTGTCGTCGCCGTCGAT |  |
| GmSWP73A-Q-RP | TCCATCGGTCTCAAAGGCGAG |  |
| GmSWP73B-Q-FP | GGGTGTCGTCACCGTTGATTTC |  |
| GmSWP73B-Q-RP | TCCACTGGTCTCAATGGGGAG |  |
| GmSYD1-Q-1F | GAAAACCTGCCATGCCTTTCAAG |  |
| GmSYD1-Q-1R | TGTAGTTTCTTCGGTGCTAGACCA |  |
| GmSYD2-Q-FP | TGGAGGCTGCTAAGTTTCTGCA |  |
| GmSYD2-Q-RP | ATAATCAGGCGCTCCACCAC |  |
| GmLFR1-Q-F | CTCCTTTGCCGATCAGAACAATA |  |
| GmLFR1-Q-R | CGCCAATCATCTATAACTTGAAGGA |  |
| GmLFR2-Q-F | AGAAGCCCAACTAAGACC |  |
| GmLFR2-Q-R | AGAGCAGCGTAAGTGTATT |  |
| GmMINU1-Q-1F | ATGGAGAATGAGCGCCAC |  |
| GmMINU1-Q-1R | ACTCACATCAGACCGAACCTT |  |
| GmMINU2-Q-1F | ATGGAGAAGGAGAATGAGCTCC |  |
| GmMINU2-Q-1R | TGCCTGTCAGGATATGCACACTT |  |
| GmMINU3-Q-1F | ATGGAGCAAGCAGTGTCACTGA |  |
| GmMINU3-Q-1R | ATACAGCGGACGACGCAA |  |
| GmBRD1-Q-FP | GCGACACAATTCAACCAACGTTGTT |  |
| GmBRD1-Q-RP | TGCGAACCATGTTGTTTACTGTC |  |
| GmBRD2-Q-FP | TCCCACAACAACAAGAACAAGACCA |  |
| GmBRD2-Q-RP | TCCCTGCGAACCATGTTTACTG |  |
| GmBRD13A-Q-FP | CGGGAAGAAGAAGAAAACGAAAC |  |
| GmBRD13A-Q-RP | AATTGCTCTAAGGTGGTATAAGATCCA |  |
| GmBRD13B-Q-FP | ACAAGTAATGCTCCTGCAGTAGAGT |  |
| GmBRD13B-Q-RP | GATGTACTTTCAGGTGATGAACCAG |  |
| GmBRD13C-Q-FP | CGAGACTGCAACGAACCACAC |  |
| GmBRD13C-Q-RP | AGCTCCTGAAACAGAAACATTAACAG |  |
| GmBRD13D-Q-FP | CGCATCGCAGCCTGATTCTC |  |
| GmBRD13D-Q-RP | ACCGGAGCTCCTGAAACAG |  |
| GmLFR1-pUBI-FP | CCTACTAGTGGATCCGGTACCATGCTGAAGAGAGAGCAAGGC | Constructs to pUBI |
| GmLFR1-pUBI-RP | GTCCTTATAGTCCATGGTACCCATACCCCATATGCCACGAGC |  |
| GmLFR1-p1300-FP | GAGAACACGGGGGACTCTAGAATGCTGAAGAGAGAGCAAG | Constructs to pCAMBIA1300 |
| GmLFR1-p1300-RP | GCACCGTATACCCCATACCCTAGGTACCACTCGTTCCCG |  |
